# Novel biallelic *USH2A* variants in a patient with usher syndrome type IIA- a case report

**DOI:** 10.1186/s12886-022-02353-7

**Published:** 2022-03-26

**Authors:** Su Ling Young, Chloe M. Stanton, Benjamin J. Livesey, Joseph A. Marsh, Peter D. Cackett

**Affiliations:** 1 Princess Alexandra Eye Pavilion, NHS Lothian, Edinburgh, UK; 2 Department of Ophthalmology, University of Edinburgh, Edinburgh, UK; 3 Medical Research Council Human Genetics Unit, Institute of Genetics and Cancer, University of Edinburgh, Edinburgh, UK

**Keywords:** Usher syndrome type IIA, Novel mutation, Pathogenicity, USH2A, Inherited retinal disease, Case report

## Abstract

**Background:**

Usher Syndrome is the commonest cause of inherited blindness and deafness. The condition is clinically and genetically heterogeneous, with no current treatment. We report a case carrying novel biallelic variants in *USH2A* causing progressive early adolescent onset visual and hearing impairment consistent with Usher Syndrome Type IIA.

**Case presentation:**

Our patient presented at age 13 with progressive visual field loss and hearing loss, associated with early onset of cataract in her 40s requiring lens extraction. Now 52 years old, latest best corrected visual acuity (BCVA) stands at Logmar Right Eye (RE) 0.8 and Left Eye (LE) 0.2, with significantly constricted visual fields bilaterally. She was registered partially sighted age 46. Clinical and molecular genetic assessment of the proband was consistent with a diagnosis of Usher Syndrome Type IIA. Genetic testing identified two novel *USH2A* variants, resulting in the premature termination codon p.Leu30Ter and a missense mutation p.Cys3251Tyr. Segregation analysis confirmed that these variants were biallelic in the affected case. Comprehensive in silico analysis confirmed that these mutations are the probable cause of Usher Syndrome Type IIA in this individual.

**Conclusions:**

The identification of novel mutations in *USH2A* increases the spectrum of genetic variations that lead to Usher Syndrome, aiding genetic diagnosis, assessment of patient prognosis, and emphasising the importance of genetic testing to identify new mutations in patients with undiagnosed progressive visual loss.

**Supplementary Information:**

The online version contains supplementary material available at 10.1186/s12886-022-02353-7.

## Background

Usher Syndrome is the commonest cause of combined inherited blindness and deafness with an estimated prevalence of 1 in 30,000 [1]. Usher syndrome was first described in 1858 by Albrecht Von Graefe, but was given it’s eponymous name for Charles Usher, a Scottish eye doctor who identified and described the disorder’s hereditary nature and recessive inheritance pattern in 1914. Usher Syndrome can be divided by clinical characteristics [2] into 3 types by the age of onset and severity of deafness, imbalance and visual loss. In addition to clinical variability, there is extensive genetic heterogeneity underlying the condition. To date, causative mutations in multiple genes, including *MYO7A*, *USH1C*, *CDH23*, *PCDH15*, *USH1G*, *CIB2*, *USH2A*, *GPR98*, *WHRN*, and *CLRN1* have been identified [3]. The mode of inheritance is typically autosomal recessive.

Homozygous or compound-heterozygous mutations in the gene encoding usherin (*USH2A*) on chromosome 1q41 have been implicated in the pathogenesis of non-syndromic retinitis pigmentosa (RP, OMIM 613809) and in Usher Syndrome Type IIA (OMIM 276901) [4] where RP occurs alongside hearing loss. *USH2A* covers 800 kb and has 72 exons. Multiple isoforms exist, but the full-length protein product is a 5202 amino acid (aa) protein of 580 kDa. Expression is restricted to the basement membrane of retinal photoreceptors and cochlear hair cells of the inner ear [5]. The precise function of USH2A remains unclear, but may have a role in cell development and maintenance [5, 6].

Mutations in *USH2A* are reported to cause 30–40% of Usher Syndrome Type II cases and 10 –15% of recessive RP cases [7]. There is a high degree of allelic heterogeneity, with many of the causative variants being identified in single cases [8]. In this study, we report a case carrying novel biallelic variants in *USH2A* causing progressive early adolescent onset visual and hearing impairment consistent with Usher Syndrome Type IIA.

## Case presentation

### Investigations

#### Clinical investigations

The proband was a 51-year-old female seen in our clinic following relocation from a different health board. She had a history of reduced vision and hearing loss since childhood, and now undergoes annual ophthalmological evaluation. This included assessment of best-corrected visual acuity (BCVA) with the ETDRS LogMAR chart, wide-field retinal imaging and red-free wide-field imaging (Optomap P200, Optos Plc, Marlborough, MA 01752,USA), spectral-domain OCT (Spectralis HRA + OCT 5.1.2.0 system; Heidelberg Engineering, Heidelberg, Germany) and electrodiagnostic testing by electroretinogram (ERG) and multifocal ERG at the Tennent’s Insitute of Ophthalmology, Glasgow.

### Genetic investigations

The proband was referred for genetic testing at the Genomic Diagnostics Laboratory within Manchester Centre for Genomic Medicine (MCGM, Manchester, UK) in 2019. Genomic DNA was isolated from peripheral blood. Next generation sequencing using an inherited retinal disease gene panel covering 175 genes was performed as previously described [9], with assessment of identified genetic variants performed by NHS Clinical scientists within the Genomic Diagnostics Laboratory. Segregation analysis was performed using a DNA sample from an unaffected child of the proband. Informed consent was obtained and the study was performed according to the principles of the Declaration of Helsinki.

### Variant interpretation

Initial interpretation of genetic variants was performed by UK National Healthcare Service (NHS) clinical scientists within the Genomic Diagnostics Laboratory at MCGM as previously described [9]. Briefly, this included assessment of the proband’s clinical referral, evaluation of scientific literature including Human Gene Mutation Database (HGMD; www.biobase-international.com/hgmd) and Genome Aggregation Database (gnomAD; https://gnomad.broadinstitute.org), and in silico prediction of pathogenicity using the Variant Effect Predictors (VEP) SIFT, PolyPhen2, MutationTaster and AlignGVGD embedded in Alamut Visual Version 2.6 (Interactive Biosoftware, Rouen, France).

Sequence conservation was determined by aligning homologous protein sequences (https://www.ncbi.nlm.nih.gov/homologene/66151) using ClustalOmega (www.ebi.ac.uk/Tools/msa/clustalo/) to identify conserved amino acid residues. Conservation scores for p.Cys3251 were calculated using PhyloP and PhastCons.

Further assessment of p.Cys3251Tyr was performed using a recently established pipeline [10] to obtain the results from 32 different VEP tools (shown in Supplementary Table 1) and comparing the predictions for 102 pathogenic and likely pathogenic USH2A missense variants from ClinVar (shown in Supplementary Table 2), and 3239 putatively benign USH2A missense variants observed in the human population from gnomAD v2.1 (shown in Supplementary Table 3).

## Results

### Clinical findings

**Fig. 1:**
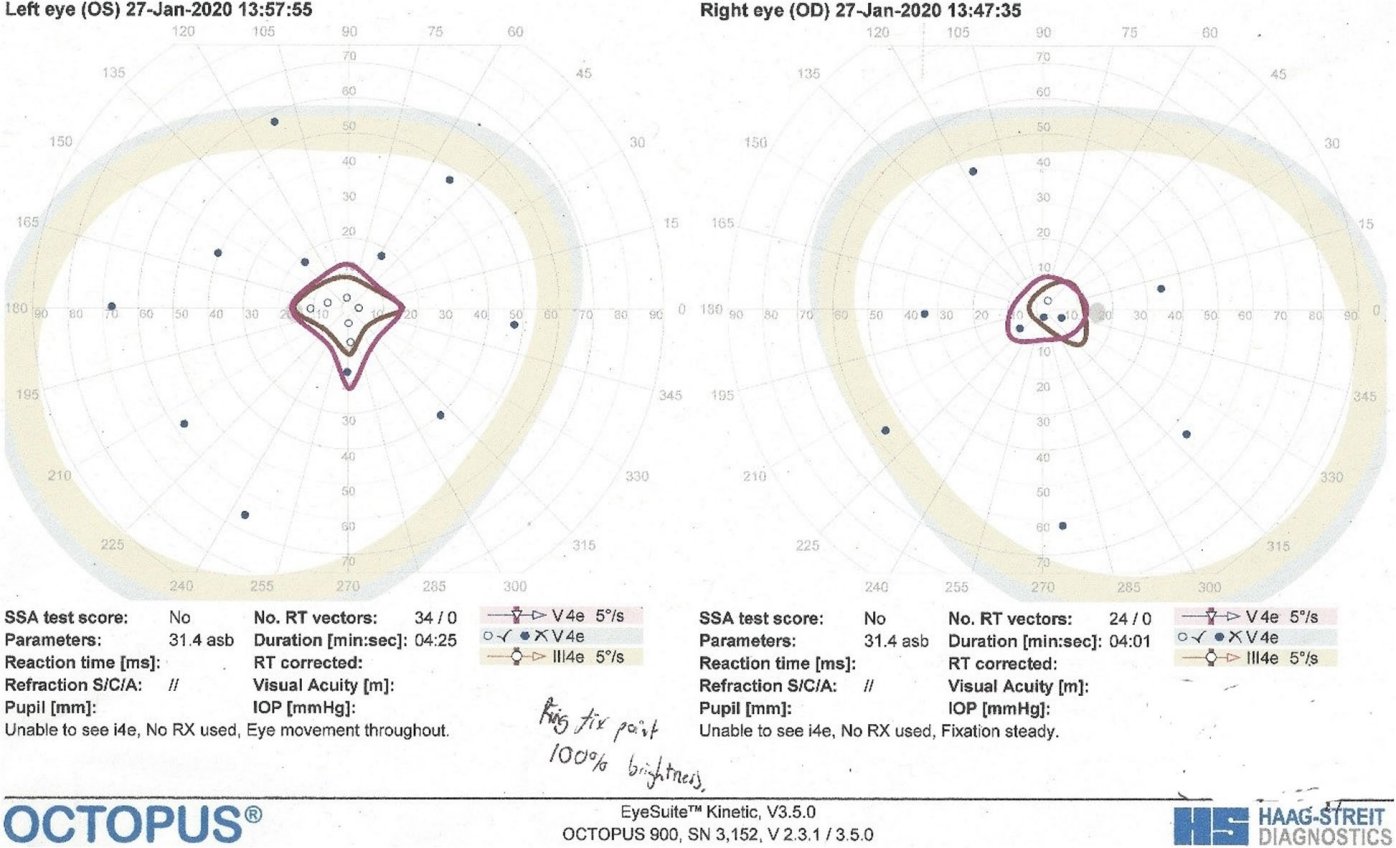
OCTOPUS® Visual Fields showing severely constricted bilateral fields, with only central island of field preserved in both eyes. In this kinetic perimetry study with stimuli V4e and III4e (indicating the largest and brightest light stimuli settings), there is significant constriction of visual fields bilaterally. In this study, both bilateral vertical and horizontal field extends no further than 20 degrees from fixation in all 4 quadrants. This is stark comparison to normal visual fields which extends 90 degrees temporally to central fixation, 50 degrees superiorly and nasally, and 60 degrees inferiorly (indicated by shaded outline)

Our patient was diagnosed with RP aged 13 following complaints of nyctalopia and reduced vision. She subsequently developed progressive visual field loss in her late teens, and underwent right phacoemulsification and intraocular lens (IOL) implant for a visually significant cataract at age 43. More recently a lamellar macular hole was noted in her right eye. Her hearing deficits began in early childhood and hearing loss was recorded at age 4. Her care was transferred to us when she relocated from a different health board. She was registered partially sighted at 45 years old. Currently at age 52, her BCVA stands at Logmar Right Eye 0.8 and Left Eye 0.2, with a lamellar macular hole in her pseudophakic right eye. Visual fields at last check-up review in January 2020 demonstrate severely constricted fields bilaterally with reduced sensitivity as shown in Fig. 1.

**Fig. 2:**
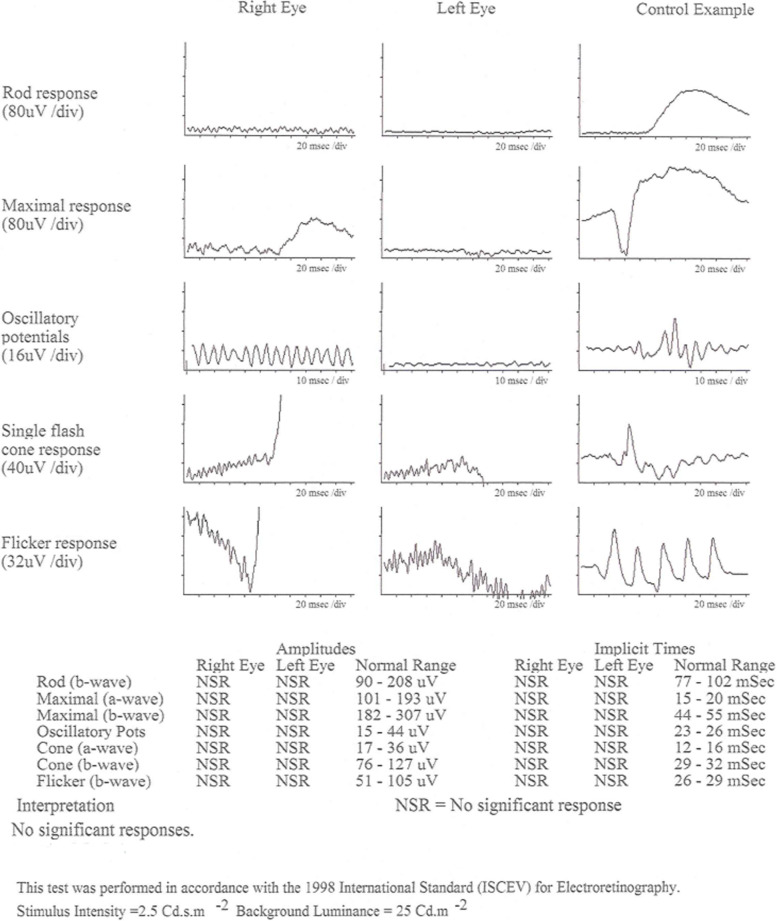
ERG report demonstrating no significant responses. In this ISCEV Ganzfeld report, ERG responses from the right and left eye are presented alongside with a control example. In both eyes (left worse than right), there are diminished responses to tests for rod, maximal response, oscillatory potentials, single flash (cone) response and flicker responses compared to the control. Analysis of amplitude and implicit times from testing did not show any significant response to stimuli (normal ranges indicated in brackets), indicating severely diminished retinal function

Electroretinography performed in 2010 (Fig. 2) demonstrate significantly reduced cone and rod responses, with significant impairment of rod,cone, maximal response, oscillatory potential and flicker responses on testing using the ISCEV Ganzfeld ERG protocols.

**Fig. 3:**
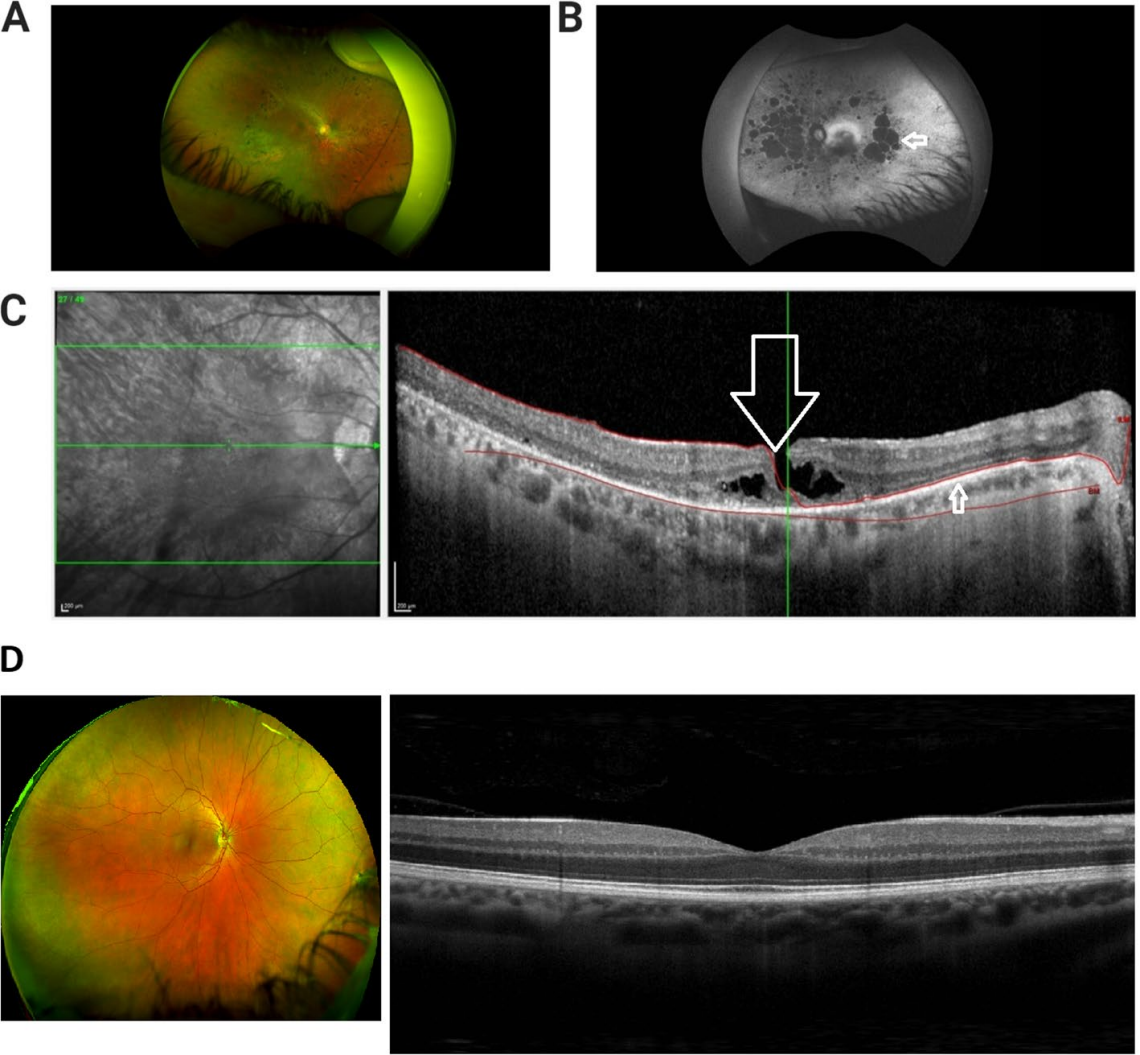
Multi-modal retinal imaging consistent with diagnosis of retinitis pigmentosa. **A**. Wide field retinal photograph depicting mid peripheral bone spicule pigmentation and perivascular atrophy. Superonasal artifact from finger holding up eyelid, eyelashes from lower lid. **B**. Red-free wide-field photograph demonstrating widespread areas of chorio-retinal atrophy in the left eye, seen as hypofluorescent /dark patches (indicated by arrow). **C**. OCT image of Right Macula depicting lamellar macular hole (indicated by large arrow). There is disruption of the inner retinal layers, however the thinned retinal pigment epithelium is intact (smaller arrow). **D**: Control wide field retinal photograph^a^ (Optos, 2022. *Healthy*. [image] Available at: <https://recognizingpathology.optos.com/healthy-retina-adult/> [Accessed 9 February 2022]) and OCT macula of normal human eye for comparison^b^ (Stoney Creek Eye Care, 2022. *Normal OCT Macula*. [Digital image] Available at: https://stoneycreekeyecare.com/what-is-optical-coherence-tomography-oct/> [Accessed 9 February 2022])

Multi-modal retinal imaging revealed bilateral mid-peripheral bone spicule pigmentation by wide-field retinal imaging (Fig. 3A) and areas of atrophy in left eye by Red-free wide-field imaging (Fig. 3B). OCT imaging demonstratess retinal pigment epithelial thinning, with a lamellar macular hole in the right eye (Fig. 3C). The ocular clinical appearances are consistent with RP.

### Genetic testing

Next generation sequencing using an inherited retinal disease gene panel covering 175 genes revealed two heterozygous mutations in *USH2A*, NM_206933.2 c.89 T > A and c.9752G > A, resulting in the premature termination codon p.Leu30Ter and a missense mutation p.Cys3251Tyr. Segregation analysis was performed using a DNA sample from an unaffected child of the proband, and confirmed that only one *USH2A* variant was inherited by the offspring of the affected individual. Biallelic variants in *USH2A* are associated with autosomal recessive Usher syndrome Type II, consistent with the diagnosis reached by clinical evaluation which included progressive retinal degeneration and hearing loss apparent at a young age.

Neither of these variants have been previously reported in scientific literature, or identified in the gnomAD database. Initial assessment of variant pathogenicity was performed using VEP tools SIFT, PolyPhen2, MutationTaster and AlignGVGD. *USH2A*, NM_206933.2 c.89 T > A, p.Leu30Ter is a nonsense variant in exon 2. This introduces a premature termination codon that is predicted to result in nonsense mediated decay (NMD) of the *USH2A* transcript, resulting in loss of expression of the encoded USH2A protein, also known as usherin. This led to the exon 2 variant *USH2A*, NM_206933.2 c.89 T > A, p.Leu30Ter being characterised as a likely pathogenic variant, contributing to Usher Syndrome in the proband.

**Fig. 4:**
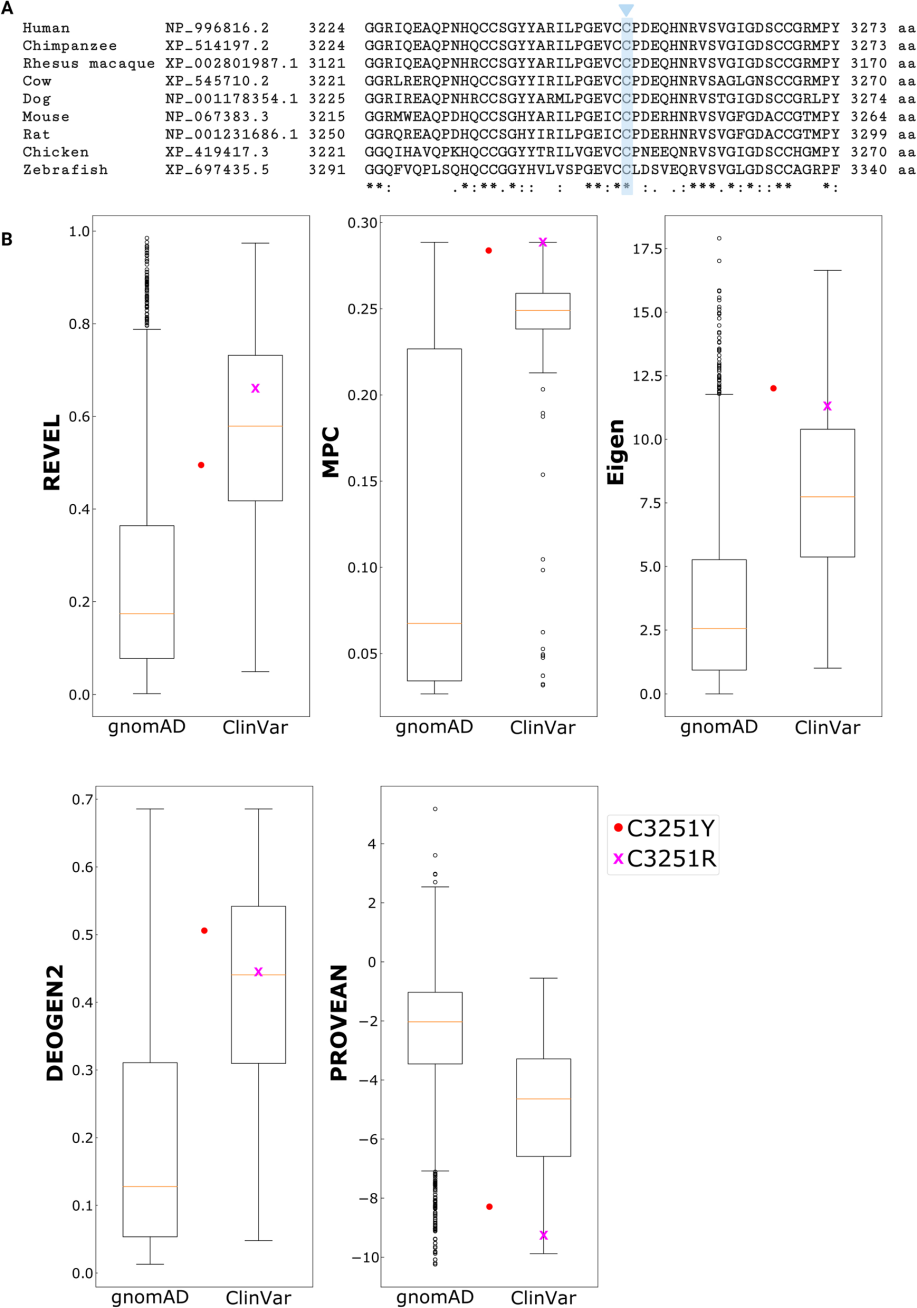
The novel missense variant p.Cys3251Tyr affects a conserved amino acid and predicted to be pathogenic. **A**. Sequence conservation of p.Cys3251 in orthologous USH2A protein sequences aligned using ClustalOmega. Cys3251, shown in blue, is completely conserved (*) throughout mammals, birds and fish. **B**. Comparison of VEP scores for 3239 putatively benign missense variants observed in the human population, taken from gnomAD v2.0, and 102 pathogenic and likely pathogenic missense variants from ClinVar. The top-five performing VEPs are included here, and the performance of all 32 tested predictors is summarized in Supplementary Table 1. The predictions for the novel p.Cys3251Tyr mutation is shown in red, and clearly clusters with the known pathogenic variants for all predictors

The second novel variant, in exon 50 of *USH2A*, NM_206933.2 c.9752G > A results in a missense change in the USH2A protein, p.Cys3251Tyr. Alignment of homologous protein sequences shows that cysteine at amino acid position 3251 is highly conserved across species (Fig. 4A), with a PhyloP score of 1.62, and a PhastCons score of 1. Initial VEP analysis indicated that substitution of cysteine for the amino acid tyrosine is likely to be damaging to the protein. However, there is no protein structure for USH2A and the amino acid substitution cannot be structurally modelled so the exact consequences of this mutation were unclear. This led to this mutation being described as a variant of uncertain significance.

Given that cysteine at amino acid position 3251 has previously been reported to be mutated to arginine in a family with Usher syndrome (p.Cys3251Arg [11];, we sought to compare the predicted pathogenicity of the novel missense change p.Cys3251Tyr to p.Cys3251Arg (Supplementary Table 1), and to other known pathogenic missense variants in *USH2A* reported in the ClinVar database to aid novel variant interpretation (Fig. 4B). First, the outputs of 32 different VEP tools were assessed for their ability to discriminate between known pathogenic and putatively benign missense variants in USH2A by calculating the area under the curve (AUC) from receiver operating characteristic (ROC) plots (Supplementary Table 1). The top performing predictors - REVEL, MPC, Eigen, DEOGEN2 and PROVEAN - were selected for further analysis of p.Cys3251Tyr. Interestingly, for all five predictors, this mutation is clearly more similar to the pathogenic ClinVar variants than the gnomAD variants, and is very similar in its predicted effects to p.Cys3251Arg (Fig. 4B). Although such assessments of performance can potentially be biased if the predictor has encountered *USH2A* mutations during its training (Livesey and Marsh, 2020), one of the top predictors, PROVEAN [12], is based on an unsupervised learning approach, and therefore immune to this bias. Notably, p.Cys3251Tyr is predicted to be more damaging by PROVEAN than 98.3% of missense variants from gnomAD and 87.3% of pathogenic missense variants from ClinVar. Together, these analyses strongly predict that the novel missense variant identified in the proband behaves more like known pathogenic variants than other variants in gnomAD, indicating that this novel variant is extremely likely to be pathogenic. As confirmed by segregation analysis, each allele of *USH2A* in the affected individual therefore carries a variant that is predicted to alter protein expression, function or stability, contributing to Usher Syndrome Type II in the proband.

## Discussion

Usher Syndrome is the commonest cause of inherited combined deafness and blindness in the developed world. Visual prognosis is poor, with studies showing 100% blindness rates in Type I patients and 67% blindness rates in Type II Usher Syndrome by age 60 [13]. A multi-centre longitudinal study noted earlier decline of visual function and a higher cumulative risk of visual impairment in type IIA Usher patients than those without non-syndromic RP, with most *USH2A* type retinitis pigmentosa patients demonstrating severe visual impairment by age 50 [14]. Our patient reported developing nyctalopia and progressive deterioration in visual fields from early adolescence, and she was registered partially sighted at 45 years old.

An increased prevalence and incidence of cataract has been reported in Usher Syndrome patients, with a prevalence of approximately 70% in Type II Usher and 80% with Type I by the age of 50 years [13]. Of the different morphological types of cataract, combined posterior subscapsular and cortical cataract are most frequently encountered in RP patients. Glare is often major symptom, prompting cataract surgery at a younger age. Our patient was recorded to have bilateral cataracts and underwent right phacoemulsification and intraocular lens (IOL) implant aged 43. A study of 142 eyes of 89 RP patients undergoing cataract surgery at one institution had noted a mean age at surgery of 47.5 years compared to a mean age at surgery of 72.5 years for non RP age-related cataract [15]. Therefore, the clinical process of cataract in our patient was not dissimilar to the presentation in other RP patient groups.

Macular pathologies have been reported in retinitis pigmentosa with a prevalence of at least 7.4%. In RP, cystoid macular oedema is most commonly reported, however macular holes and epiretinal membranes are also known associations [16, 17]. Our patient also developed a partial thickness macular hole in her right eye which was diagnosed at 44 years old. Whilst surgical intervention (pars plana vitrectomy) can improve morphological appearances, visual gain is often limited by co-existing retinal dysfunction [16].

To date there are no therapeutic options for Usher Syndrome, but uncovering causative genes and pathogenic variants therein provides biological insights into the disease process and has important implications for disease prognosis. Mutations in *USH2A* are reported to cause 30–40% of Usher Syndrome Type II cases and 10 –15% of recessive RP cases [7]. There is substantial genetic heterogeneity even within RP and Usher Syndrome caused by variation in *USH2A*, with 606 pathogenic or likely pathogenic variants distributed throughout the gene deposited in ClinVar to date (accessed September 2020). Our study adds to the increasing number of *USH2A* pathogenic or likely pathogenic variants that have been identified. Molecular consequences of the ClinVar variants include 174 frameshift mutations, 99 missense mutations, 96 affecting splice sites, and 165 nonsense mutations. Many of these variants affect only one individual, but when considered together will lead to a better understanding of genotype-phenotype correlations in non-syndromic RP and Usher Syndrome Type II.

Individuals with Usher Syndrome carrying two truncating variants in *USH2A* have a younger age of hearing loss, and progress more rapidly to visual impairment and legal blindness compared to patients in which one or two alleles encode missense changes [18]. This is consistent with earlier studies, showing that residual protein function may lead to non-syndromic RP or slower progression of ocular manifestations. Conversely, Usher Syndrome Type II is most likely to arise as a result of a complete loss of function of USH2A [14]. Predicting the impact of missense changes in USH2A is complicated by the variability in performance of VEP tools (Supplementary Table 1). VEP utilise combinations of sequence conservation, known genetic variation, epigenetic modification, functional annotation of the protein, and available protein structural information to predict how a mutation may affect protein expression, stability and function. In addition to this, many *USH2A* variants occur as compound heterozygotes, as is the case for our proband, where different mutations in *USH2A* are inherited from maternal and paternal chromosomes. A better understanding of the impact of missense mutations on USH2A is crucial, and the comprehensive in silico assessment of pathogenicity performed in our study is an important step towards this goal. This will contribute towards dissecting the genetic and clinical heterogeneity of *USH2A*-associated RP and Usher Syndrome Type II*,* improving clinical management of Usher Syndrome by aiding appropriate genetic counselling and prognosis of disease progression.

## Conclusions

This report highlights two novel variants in *USH2A* associated with Usher Syndrome Type II, with associated visual implications. We hope that these novel mutations may contribute to genotype-phenotype studies, and inform clinical management of Usher Syndrome by aiding appropriate prognosis of disease progression and genetic counselling. *USH2A* is the subject of ongoing research towards treatment modalities including use of antisense oligonucleotides targeting exon 13 (QR-421a, Phase1/2 clinical trials, ProQR, patent no. US 10,131,910 B2), but the distribution of pathogenic variants throughout the gene, together with the size of the gene and the protein it encodes, mean that a greater understanding of causative mutations is required for all patients to be treatable in the future. For the clinician, we wish to encourage thinking of new causative genetic variants in patients with undiagnosed progressive visual loss and to consider re-discussion with genetics colleagues and repeat genetic testing.

### Learning points

- The identification of novel mutations in *USH2A* increases the spectrum of genetic variations that lead to Usher Syndrome.
- We hope that these novel mutations may contribute to genotype-phenotype studies and inform clinical management by aiding genetic counselling and prognostication of disease progression.
- For the clinician, we would like to encourage thinking of new gene variants and to consider repeat genetics testing in evaluation of patients with undiagnosed progressive visual loss.

## Supplementary Information

**Additional file 1.**

**Additional file 2.**

**Additional file 3.**

## Data Availability

All data generated or analysed during this study are included in this published article [and its supplementary information files.]

## Abbreviations

USH2A: Usher syndrome 2A
BCVA: Best corrected visual acuity
RE: Right eye
LE: Left eye
MYO7A: Myosin VIIA
USH1C: USH1 Protein Network Component Harmonin
CDH23: Cadherin Related 23
PCDH15: Protocadherin Related 15
USH1G: USH1 Protein Network Component Sans
CIB2: Calcium And Integrin Binding Family Member 2
GPR98: Adhesion G Protein-Coupled Receptor V1
WHRN: Whirlin
CLRN1: Clarin 1
RP: Retinitis pigmentosa
Kb: kilobase
Aa: amino acid
kDa: kilodalton
OCT: Optical coherence tomography
ERG: Electroretinogram
NHS: National Healthcare Service
MCGM: Manchester Centre for Genomic Medicine
HGMD: Human Gene Mutation Database
gnomAD: Genome Aggregation Database
VEP: Variant Effect Predictor
IOL: Intraocular lens
AUC: Area under the curve
ROC: Receiver operating characteristic
